# Odon device: a promising tool to facilitate vaginal delivery and increase access to emergency care

**DOI:** 10.1186/1742-4755-10-42

**Published:** 2013-08-20

**Authors:** Jennifer Harris Requejo, José M Belizán

**Affiliations:** 1 Institute for International Programs, Johns Hopkins Bloomberg School of Public Health, Baltimore, Maryland, USA; 2 Institute for Clinical Effectiveness and Health Policy, Buenos Aires, Argentina

## Abstract

The last innovation in operative vaginal delivery happened centuries ago with the invention of the forceps and the vacuum extractor. The World Health Organization Odon Device Research Group recently published a protocol for a feasibility and safety study for a new device (Odon device) which aims to revolutionize assisted vaginal delivery. This editorial discusses the device and its pathway to global use. Although preliminary results look promising, the rigorous three-phased WHO protocol needs completion before the device can be determined, based on the evidence, to be safe and effective.

## 

In this journal, the World Health Organization Odon Device Research Group describes the protocol for a feasibility and safety study of a new device (Odon device) for assisted vaginal deliveries [1]. This device is a low cost, easy to use technological innovation to facilitate operative vaginal delivery when complications occur during the second stage of labor. It is designed to minimize trauma to the mother and the baby, and may be possible for application by mid-level providers. These features combined make the Odon device a potentially revolutionary development in obstetrics. As the article points out, the last innovation in operative vaginal delivery happened centuries ago with the invention of the forceps and the vacuum extractor, both of which require a high level of skill to use and are associated with certain risks of injury to mother and baby. Similarly, caesarean delivery – a third option for clinical management of prolonged second stage of labour – is out of reach for many women living in low resource settings, making innovations like the Odon device essential for increasing women’s access to needed obstetrical care.

Complications related to prolonged second stage of labor are responsible for countless women’s and children’s deaths and long-term disabilities [2,3]. The Odon Device represents a promising novel way to potentially prevent these deaths and improve obstetrical outcomes for both mother and baby. Given its simplicity and the likelihood that mid-level providers can be trained on its use, the device could increase multi-fold women’s access to quality emergency obstetrical care – particularly the most vulnerable populations who struggle to reach tertiary care facilities when something goes wrong during childbirth. The possibility that use of the device could have additional benefits such as counteracting the worrisome rise in caesarean deliveries worldwide, and reducing perinatal infections acquired through the birth canal during childbirth must also be explored.

## The seeds of innovation – the Odon device’s humble origins

The story of the development of the Odon Device is a tribute to human ingenuity, and gives further credence to Albert Einstein’s adage, “imagination is more important than knowledge”. In 2005, Mr. Jorge Odon, a car mechanic from Argentina with no medical training, was inspired to design the device after thinking carefully about the basic scientific principles underlying a simple trick he used to perform for his friends. This trick involved removing a cork from the inside of an empty bottle without breaking it. His eureka moment happened when he realized that the solution to the cork challenge could be adapted to a method for facilitating childbirth.

What followed this initial spark was a rapid uptake of the idea from Mr. Odon’s first collaboration in 2006 with the Center for Medical Education and Clinical Investigation (CEMIC) in Buenos Aires, Argentina and professionals from WHO, to the testing of the device in 2008 on a simulator at Des Moines University, Iowa, USA, to the approval in 2009 of a World Health Organization study protocol to test the device on human beings, and, finally, to the awarding in 2011 of a “Saving Lives at Birth: A Grand Challenge for Development” grant to further test the potential of the device to save women’s and children’s lives at the time of birth when the majority of maternal and newborn deaths occur.

## Where are we now? The pathway to global use

The World Health Organization Odon Device Research Group’s article in this journal describes in detail the first phase of the WHO’s three-phased study protocol aimed at bringing the Odon Device from a clever, long-overdue idea into the ‘obstetric arsenal’ – expanding the clinical management tools available to practitioners for assisted vaginal delivery. The first phase is underway and involves the testing of the device under normal delivery conditions in tertiary hospitals in Argentina and South Africa. The next two phases will assess preliminary efficacy in facilitating delivery when women are experiencing prolonged second stage of labor with no fetal distress, and compare the effectiveness of the device to the vacuum extractor and forceps.

Although preliminary results look promising, the rigorous three-phased WHO protocol needs completion before the device can be determined, based on the evidence, to be safe and effective.

## References

[B1] The World Health Organization Odon Device Research Group Feasibility and safety study of a new device (Odón device) for assisted vaginal deliveries: study protocol Reprod Health 2013 10 33 23822879 10.1186/1742-4755-10-33 PMC3707820

[B2] AbouZahr C Rodeck C Global burden of maternal death and disability Reducing maternal death and disability in pregnancy 2003 Oxford: Oxford University Press 1 11

[B3] Ronsmans C Graham WJ on behalf of The Lancet Maternal Survival Series steering group Maternal mortality: who, when, where, and why Lancet 2006 368 1189 1200 10.1016/S0140-6736(06)69380-X 17011946

